# Evaluation of the “RehabXR” virtual reality system for vestibular rehabilitation: evidence of changes in functional brain connectivity in warfighters with chronic mild traumatic brain injury

**DOI:** 10.3389/fnhum.2026.1850913

**Published:** 2026-06-05

**Authors:** Zhihao Li, Carrie W. Hoppes, Sridhar Ramakrishnan, Pedram Hovareshti, Pearson Van Horn, Matthew DiSalvo, Lisa S. Holt, Shane R. Salter, Holly Richard, Shannon L. Barnicott, Meghan T. Logeais, Michael D. Wirt, Susan L. Whitney

**Affiliations:** 1 AeroVironment, Inc. (Formerly, BlueHalo, LLC.), Germantown, MD, United States; 2 Advanced Exposures, Diagnostics, Interventions, and Biosecurity (AEGIS) Program, Joint Base San Antonio – Lackland Air Force Base, TX, United States; 3 Department of Rehabilitation Medicine, Brooke Army Medical Center, Center for the Intrepid, Joint Base San Antonio - Fort Sam Houston, TX, United States; 4 U.S. Department of Veterans Affairs, South Texas Veterans Health Care System, San Antonio, TX, United States; 5 Department of Physical Therapy, University of Pittsburgh, Pittsburgh, PA, United States

**Keywords:** functional connectivity, military service members, resting-state functional magnetic resonance imaging, vestibular function, virtual reality rehabilitation

## Abstract

**Introduction:**

Military service members (SMs) often experience vestibular dysfunction, including symptoms like dizziness and gaze instability, following mild traumatic brain injury (mTBI). These impairments compromise their ability to perform military duties, emphasizing the necessity for effective rehabilitation strategies. We previously developed a mixed reality-based system, called RehabXR, to deliver multisensory vestibular rehabilitation using low-cost wearable sensors and a virtual reality environment.

**Methods:**

This study evaluates RehabXR’s rehabilitative impact both behaviorally and at neural level in 15 SMs with chronic vestibular complaints post-mTBI. Behavioral assessments were conducted via a Readiness Assessment Battery comprising mobility and agility tests, while neural impact was measured using resting-state functional MRI (rfMRI) to evaluate functional connectivity (FC) in the vestibular visual network (VVN) and default mode network (DMN).

**Results:**

After the rehabilitation, participants showed significant improvements in task completion times, as well as increased FC in the VVN and decreased FC in the DMN.

**Discussion:**

These findings suggest RehabXR as a promising rehabilitative tool for improving vestibular function and cognitive-motor integration post-mTBI.

## 1 Introduction

Symptoms of vestibular dysfunction, such as dizziness, gaze instability, disorientation, and cognitive fog, are prevalent in military service members (SMs) after mild traumatic brain injury (mTBI) (Swan et al., 2020). These symptoms compromise routine military tasks (e.g., ruck marching and obstacle course navigation), so there remains a critical need for intervention and associated evaluation of effectiveness. Beyond their immediate functional impact, these persistent complaints are increasingly understood as manifestations of longer-term neurobiological changes, including transcriptomic adaptations (e.g., alternative splicing and gene-expression shifts) that overlap with patters seen across a broader neurodevelopmental-neurodegenerative continuum (Kuang et al., 2023; Ran et al., 2026), as well as cortical remodeling that reweights large-scale network organization (Smith et al., 2022). Within this framework, mTBI is not merely a transient insult but a trigger for prolonged brain plasticity, providing a mechanistic rationale for why targeted, multisensory vestibular rehabilitation might redirect these large-scale networks toward more adaptive configurations over time. Previous efforts to leverage this plasticity have yielded either a high-cost and large rehabilitation environment, such as the Computer-Assisted Rehabilitation Environment (CAREN) system (Motech Medical, Houten, Netherlands) (Gottshall et al., 2012); or a low-cost and portable device, such as the VestAid (AeroVironment, Germantown, MD) (Hovareshti et al., 2021). However, despite these advances, existing solutions have yet to achieve an optimal balance among cost, portability, and ecological validity in the specific context of military tasks.

**Figure 1:**
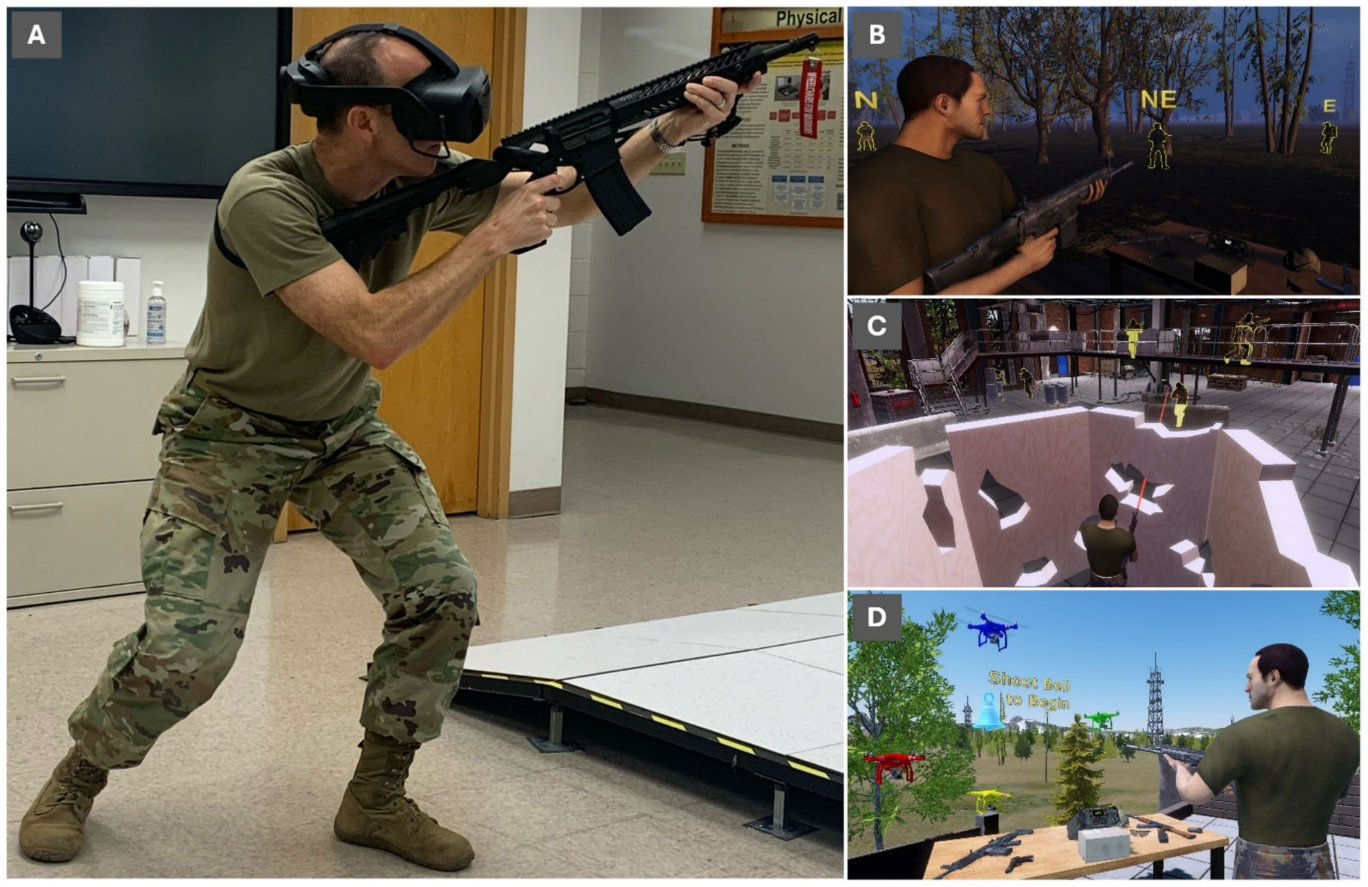
The RehabXR system **(A)** delivers first-person-shooter serious games of directional memorization **(B)** (shoot enemies in response to directional cue), barricade wave defense **(C)** (shoot enemies from behind a barricade), and Stroop target shoot **(D)** (shoot drones matching the color of the cue). In an environment of mixed reality, these games challenge a player on spatial and verbal memory, attention, response inhibition, smooth pursuit, saccades, gaze stability, agility, dynamic stability, and response inhibition.

To fill this gap, we previously developed a portable mixed reality based (RehabXR, formerly known as Praxis) system, incorporating low-cost wearable sensors and a virtual reality environment for delivering multisensory vestibular rehabilitation to SMs with persistent symptoms after mTBI (Alroumi et al., 2025). RehabXR provides three first-person shooter serious games for enhancing gaze stability, balance, and cognitive-motor integration (Figure 1). Correspondingly, assuming effectiveness, the rehabilitative impact of these physical and cognitive exercises is expected to manifest in the brain as the potential biomarker. The present study aims to evaluate the rehabilitative effect of RehabXR both in the behavioral and neural level. In the behavioral level, we currently focus on a Readiness Assessment Battery (see more details in the Methods) for evaluating functional changes on mobility, agility, speed, and endurance. In the neural level, we use resting-state functional magnetic resonance imaging (rfMRI) (Morelli et al., 2021) to access functional connectivity (FC) in associated brain networks.

In the context of mTBI and vestibular rehabilitation, two functional brain networks are of specific interest: the vestibular visual network (VVN) and the default mode network (DMN). The VVN is a multisensory and task-positive network for integrating vestibular, visual, and somatosensory inputs to support balance, spatial orientation, and visual motion processing (Lopez and Blanke, 2011). The DMN, on the contrary, is a task-negative network associated with self-referential thought, mind-wandering, and internal mentation (Raichle, 2015). In healthy individuals, these two networks typically exhibit antagonistic interactions with the DMN activity being suppressed during tasks that engage external sensory integration and attention (Fox et al., 2005). Previous studies have suggested a breakdown in the dynamic balance between the task-positive and task-negative networks in mTBI, where impaired sensory integration due to VVN dysfunction may lead to insufficient suppression, or even hyperactivity, of the DMN (Gilmore et al., 2016; Sours et al., 2013). This aberrant interplay between sensory and default-mode networks may contribute to the co-occurrence of perceptual and cognitive symptoms seen in patients with mTBI (Palacios et al., 2013). Therefore, FC changes in these two networks are likely biomarkers in the RehabXR rehabilitation. Because of this rehabilitative training, we expected improvements on visual-vestibular integration as measured by the Readiness Assessment Battery; and because this assessment battery engages external inputs and attention, we hypothesized an increased FC in the VVN and decreased FC in the DMN.

## 2 Methods

### 2.1 Subjects

Subjects for this study were recruited from the Center for the Intrepid’s Special Operations Performance and Recovery (SPaR) Program at Brooke Army Medical Center. Recruitment flyers posted at the Center for the Intrepid (CFI), word of mouth, and recruitment briefings were used to advertise the study. At the end of the first day of the 4-week SPaR Program, a member of the study team not involved in the rehabilitation of the potential participants (e.g., study personnel not assigned to the CFI as staff) provided a recruitment briefing. Interested individuals who provided written informed consent were then assessed against the study’s inclusion and exclusion criteria for assignment to the RehabXR or the Control group.

The RehabXR group consisted of 15 SMs (all male, age mean ± std. = 37.0 ± 5.0 y.o.) with a self-reported history of mTBI and unresolved vestibular-related complaints They completed two behavioral test sessions with the Readiness Assessment Battery and two rfMRI sessions at baseline, and again, 4 weeks later, before and after the standard SPaR Program interventions plus RehabXR training. As reported previously (Alroumi et al., 2025), the RehabXR group participated in the SPaR Program combined with 45 min of supervised RehabXR therapy daily, 5 days a week, in addition to a home exercise program (HEP) consisting of 20 min of unsupervised VestAid therapy daily, 5 days a week. VestAid is a tablet-based technology designed to support vestibular rehabilitation by monitoring and guiding patients through vestibulo-ocular reflex (VORx1) exercises (Hovareshti et al., 2021). It uses the tablet’s front-facing camera and embedded algorithms to track eye and head movements in real time, providing objective performance metrics (e.g., gaze stability and exercise adherence) hence enabling individualized home exercise for enhanced patient engagement and treatment efficacy. Over the course of this study, the RehabXR group engaged in approximately 20 training sessions of RehabXR and VestAid (5 days a week for 4 weeks).

The control group consisted of 15 healthy SMs (all male, age mean ± std. = 37.3 ± 4.8 y.o.) without vestibular-related complaints. They completed two behavioral test sessions with the Readiness Assessment Battery at baseline, and again, 4 weeks later, before and after the SPaR Program intervention without RehabXR/VestAid training. They were not evaluated using rfMRI.

### 2.2 Readiness Assessment Battery

The Readiness Assessment Battery included the (i) Hybrid Assessment of Mobility for mild Traumatic Brain Injury (HAM-4-mTBI) (Fino et al., 2023), (ii) modified version of the Portable Warrior Test of Tactical Agility (POWAR-TOTAL) (Cecchini et al., 2023; Favorov et al., 2023), and (iii) 300-yard Shuttle Run (Barringer et al., 2019; Conkright et al., 2020). This battery was administered by members of the study team not involved in the rehabilitation of the participants (e.g., study personnel not assigned to the CFI as staff). They were not blinded to group assignment.

The HAM-4-mTBI is a condensed, hybrid mobility assessment based on the Functional Gait Assessment (FGA) (Wrisley et al., 2004) and the High-Level Mobility Assessment Tool (HiMAT) (Williams et al., 2005) to monitor individuals with mTBI. Subjects completed the FGA gait with horizontal head turns and gait with pivot turn (each scored according to the FGA criteria from 0 to 3), as well as the HiMAT fast forward and backward walk (each scored according to the HiMAT criteria from 1 to 4). The HAM-4-mTBI yielded an area under the curve of 0.71 (95% confidence interval 0.61–0.79) using standard scoring for discriminating persons with persistent symptoms from mTBI from healthy controls (Fino et al., 2023).

During the modified version of the POWAR-TOTAL (Cecchini et al., 2023; Favorov et al., 2023), the subject carried a simulated standard service weapon (Bluegun M4). The subject began the test in a prone position. They then stood up, ran diagonally left/forward for 3 m, dropped back into a prone position on a floor mat and performed a clockwise combat roll. Afterward, they stood and backpedaled to the starting position, side-shuffled to the left, ran diagonally right/forward for 3 m, dropped back into a prone position on a floor mat and performed a counterclockwise combat roll, stood up and backpedaled to the starting line, and side-shuffled to the right to end at the starting position. The modified POWAR-TOTAL was performed as a single- (motor only) and dual-task (motor + cognitive). For the cognitive task, the subject was asked to recall an 8-digit grid coordinate. Concussed active-duty SMs were slower than healthy controls for single- and dual-task motor performance with moderate effect sizes and were less accurate in recalling grid coordinates in both single- and dual-task conditions with moderate to large effect sizes on the POWAR-TOTAL (Cecchini et al., 2023).

As part of the Ranger Athlete Warrior assessment battery, subjects ran 25 yards (22.86 m), turned, and ran 25 yards (22.86 m) back, completing this sequence six times for a total of 300 yards (274.32 m). The 300-yard Shuttle Run was also performed as a single- (motor only) and a dual-task (motor + cognitive). For the cognitive task, the subject was asked to recall an 8-digit grid coordinate. The 300-yard Shuttle Run was selected due to its repeated turning demands, as individuals with persisting balance-related symptoms after mTBI are known to have slower turning speeds and more variable head-on-body coordination (Fino et al., 2018).

Specific task performances analyzed in the present study are shown in Table 1. Taken together, these tasks evaluate physical performance and readiness across military and civilian populations recovering from mTBI, as well as healthy military personnel.

**Table 1:** Statistical comparison of Readiness Assessment Battery performance between the ‘start’ (baseline) and ‘finish’ (after 4 weeks of training) assessments for both the RehabXR and control groups. The group difference on the training effect is shown in the last two columns of effect size (Cohen’s f^2^) and *p* values for the “Group x Time” effect.

Readiness Assessment Battery	Task	RehabXR group				Control group				Group x time Cohen’s f^2^	Group x time *p*-value
		Start	Finish	N^*^	Paired *t*-test*p*-value	Start	Finish	N^*^	Paired *t*-test*p*-value		
modified POWAR-TOTAL	Single task motor time (in sec)	18.63	18.09	14	0.29	19.65	18.86	14	0.21	0.026	0.68
	Single task cognitive score	6.86	7.14	14	0.47	6.92	6.57	14	0.57	0.033	0.41
	Dual task motor time (in sec)	17.54	16.49	14	0.05	18.55	17.77	14	0.15	0.022	0.73
	Dual task cognitive score	5.79	6.29	14	0.49	6.07	6.07	14	1.00	0.032	0.64
HAM-4-mTBI: FGA Tasks	Gait with horizontal head turns score	1.86	2.00	14	0.61	2.86	2.71	14	0.33	-0.001	0.49
	**Gait with horizontal head turns time (in sec)**	**9.00**	**7.32**	**14**	**0.00**	**7.57**	**6.84**	**14**	**0.02**	**0.080**	**0.03**
	Gait and pivot turn score	2.07	2.36	14	0.10	2.79	3.00	14	0.08	-0.024	0.81
	Gait and pivot turn time (in sec)	5.63	4.69	14	0.00	4.61	4.14	14	0.23	0.023	0.30
HAM-4-mTBI: HiMAT Tasks	Walking forward score	3.43	3.50	14	0.79	3.71	3.93	14	0.08	-0.005	0.55
	Walking forward time (in sec)	9.47	8.96	14	0.11	8.40	7.11	14	0.11	0.042	0.31
	Walking backward score	3.50	3.57	14	0.58	3.93	3.93	14	-	-0.036	0.63
	Walking backward time (in sec)	11.45	10.97	14	0.30	9.66	8.61	14	0.21	0.038	0.46
300-yard Shuttle Run	Single task total time (in sec)	77.91	84.01	14	0.22	79.84	81.28	14	0.44	0.106	0.33
	Single task cognitive score	7.79	7.57	14	0.34	7.29	7.21	14	0.86	0.006	0.74
	Dual task total time (in sec)	85.64	84.63	14	0.69	80.18	80.90	14	0.64	0.075	0.58
	Dual task cognitive score	6.64	7.07	14	0.35	6.79	7.21	14	0.40	0.017	0.96

FGA: Functional Gait Assessment; HAM-4-mTBI: 4-Item Hybrid Assessment of Mobility for mild Traumatic Brain Injury; HiMAT: High-Level Mobility Assessment Tool; POWAR-TOTAL: Portable Warrior Test of Tactical Agility. The bold face row indicates a significant Group x Time interaction on that task. * number of subjects with data available in both the “Start” and “Finish” tests. - no results as the “Start” and “Finish” values are identical in each control subject. It leads to 0 standard deviation on the differences, so *p* values of the Time effect were incalculable.

### 2.3 MRI data acquisition

With a Siemens 3 T scanner (MAGNETOM Skyra Fit), anatomical and functional images were acquired from each RehabXR group subject in each MRI session. The anatomical scan used a magnetization prepared rapid acquisition gradient echo (MPRAGE) sequence with key imaging parameters of TR = 2,530 ms, TE = 2.6 ms, FA = 7^o^, FOV = 256×256mm^2^, Matrix = 256×256, #SagittalSlices = 176, and Thickness = 1 mm. The functional scan used an echo-planar imaging (EPI) sequence with key parameters of TR = 2,130 ms, TE = 30 ms, FA = 85^o^, FOV = 220×220mm^2^, Matrix = 64×64, #AxialSlices = 39, Thickness = 3.4 mm, #Volume = 245. During the functional scans, subjects were instructed to keep their eyes closed but remain awake.

### 2.4 MRI data analysis

**Figure 2:**
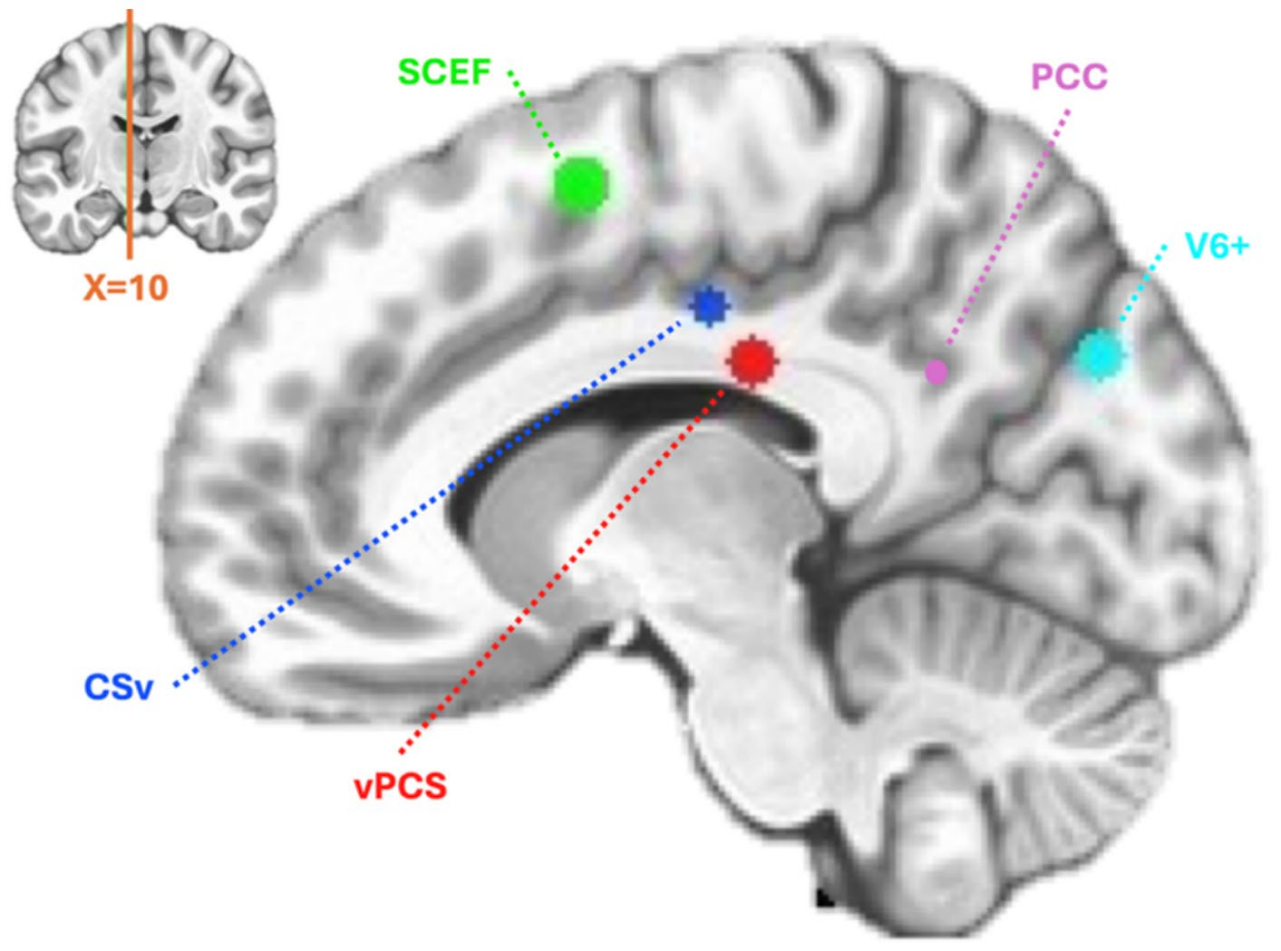
Seeding regions for probing the vestibular visual network (VVN; SCEF, CSv, vPCS, V6+) and Default Mode Network (PCC). Their Montreal Neurological Institute (MNI) coordinates and anatomical descriptions are listed in Table 2. There are 8 bilateral seeding regions for the VVN in total but only 4 are shown here in the right hemisphere. In the analysis, all seeding regions are the same size, but they are visualized in different sizes here due to the projection on a single sagittal slice with position (*X* = 10 mm) indicated in the top left coronal view.

The rfMRI data analysis pipeline was implemented with the “AFNI” software package (afni.nimh.nih.gov). At the individual level, this pipeline went through 10 preprocessing steps of (i) outlier detection, (ii) de-spiking, (iii) slice timing correction, (iv) volume registration, (v) template normalization into the Montreal Neurological Institute (MNI) space, (vi) image segmentation for gray matter, white matter, and cerebral spinal fluid (CSF), (vii) nuisance signal (head motion, white matter and CSF) regression, (viii) spatial smoothing (FWHM = 5 mm), (ix) voxel-wise seeding correlation, and (x) Fisher’s z (FZ) transformation. To probe the VVN, the seeding region in step (ix) was a combination of 8 spherical regions (*r* = 3 mm) derived from MNI coordinates previously reported on vestibular and visual brain areas (Beer et al., 2023). To probe the DMN, the seeding region coordinates were derived from a meta-analysis on “NeuroSynth”^1^ based on 907 studies and the keyword of “default.” All the seeding regions are visualized in Figure 2 with their MNI coordinates shown in Table 2.

**Table 2:** Seeding regions for probing the vestibular visual network (VVN) and default mode network (DMN).

Functional network	Seeding region	X (mm)	Y (mm)	Z (mm)	Note
VVN					
	Left vPCS	−7.3	−23.8	28.9	Vestibular pericallosal sulcus
	Right vPCS	7.5	−24.4	28.8	
	Left CSv	−13.7	−17.2	38.2	Cingulate sulcus visual
	Right CSv	14.2	−17.6	38.8	
	Left V6+	−12.5	−81.2	30.3	Area V6 and satellite
	Right V6+	16.4	−80.1	31.1	
	Left SCEF	−9.1	3.8	57.7	Supplementary (cingulate) eye field
	Right SCEF	7.6	4.6	57.2	
DMN					
	PCC	0	−52	26	Posterior cingulate gyrus

The Montreal Neurological Institute (MNI) coordinates of XYZ are in the orientation of left/posterior/inferior (LPI). These seeding regions are also visualized in Figure 2.

For noise/artifact control, individual’s preprocessed data must have satisfied three criteria (Power et al., 2012) to be included in the subsequent group-level analysis: (1) temporal datapoint censored (or scrubbed) out should be less than 20%; (2) after the temporal censoring, the remaining data should still be more than 5 min in length; and (3) the max pair-wise head motion should be less than 3 mm. Out of the total 30 imaging sessions (2 sessions/subject x 15 subjects) these thresholds excluded 7 imaging sessions with severe artifact (5 sessions), or head motion (1 session), or both (1 session), leaving 23 sessions for the subsequent group-level analysis. In these 23 sessions, the mean (across imaging sessions) framewise displacement were 0.37 mm before motion censoring versus 0.18 mm after motion censoring. Please note that the present exclusions were completely data driven and simply for the purpose of quality control. While sample attritions vary substantially in the rfMRI literature, the present exclusion rate does fall in the range of 9–36% reported by Weiler et al. when “evaluating denoising strategies” in rfMRI and TBI (Weiler et al., 2022).

After the image preprocessing at the individual level, our group-level data analysis in the RehabXR group followed with a voxel-wise linear mixed effect modeling (LMEM) of FC~SESSION+MOTION , where FC means functional connectivity (FZ values in each voxel), SESSION represents the imaging sessions of either “start” or “finish,” and MOTION represents the max head motion in an imaging session. In this LMEM, we were interested in the SESSION effect while statistically controlling the confounding effect of MOTION on changes of FC . For the correction of multiple comparisons, the clustering threshold of this LMEM result was determined by a Monte-Carlo simulation with a voxel-wise *p* value threshold of 0.001, and the noise smoothness structure taken into account by the autocorrelation function implemented in AFNI’s 3dClustSim (Cox et al., 2017).

## 3 Results

### 3.1 Behavioral performance of the Readiness Assessment Battery

On the behavioral assessments, missing data were noticed from one of the RehabXR and one of the control subjects. The control subject was withdrawn prior to this battery test as his occupational therapist advised exclusion “due to behavioral concerns that could compromise data validity or safety and potentially exacerbate an unrecognized health condition.” The RehabXR subject did perform the testing, but his data was lost due to a technical error with laptop malfunction. The sample size analyzed for the Readiness Assessment Battery was 14 subjects in each group.

Through a LMEM with fixed effects of Group (patients vs. controls) and Time (training “start” vs. “finish”) and their interactions, as well as random intercept for subjects, a significant interaction effects were observed on the task items of “HAM-4-mTBI: FGA: Gait with horizontal head turns time” (*p* = 0.03). In this task, the patients exhibited a significantly (*p* < 0.001) reduced completion time from ~9.00sec (“start) to ~7.32sec (“finish”), while the same reduction for the controls was not as much (7.57sec to 6.84sec, *p* = 0.02). Similar reductions on completion time were also noticed on “HAM-4-mTBI: FGA: Gait and pivot turn time” and “modified POWAR-TOTAL: Dual task motor time”, but the group differences on these reductions were not significant. Other comparisons on behavioral performances between the Group and Time are listed in Table 1.

### 3.2 FC changes in the VVN and DMN

**Figure 3:**
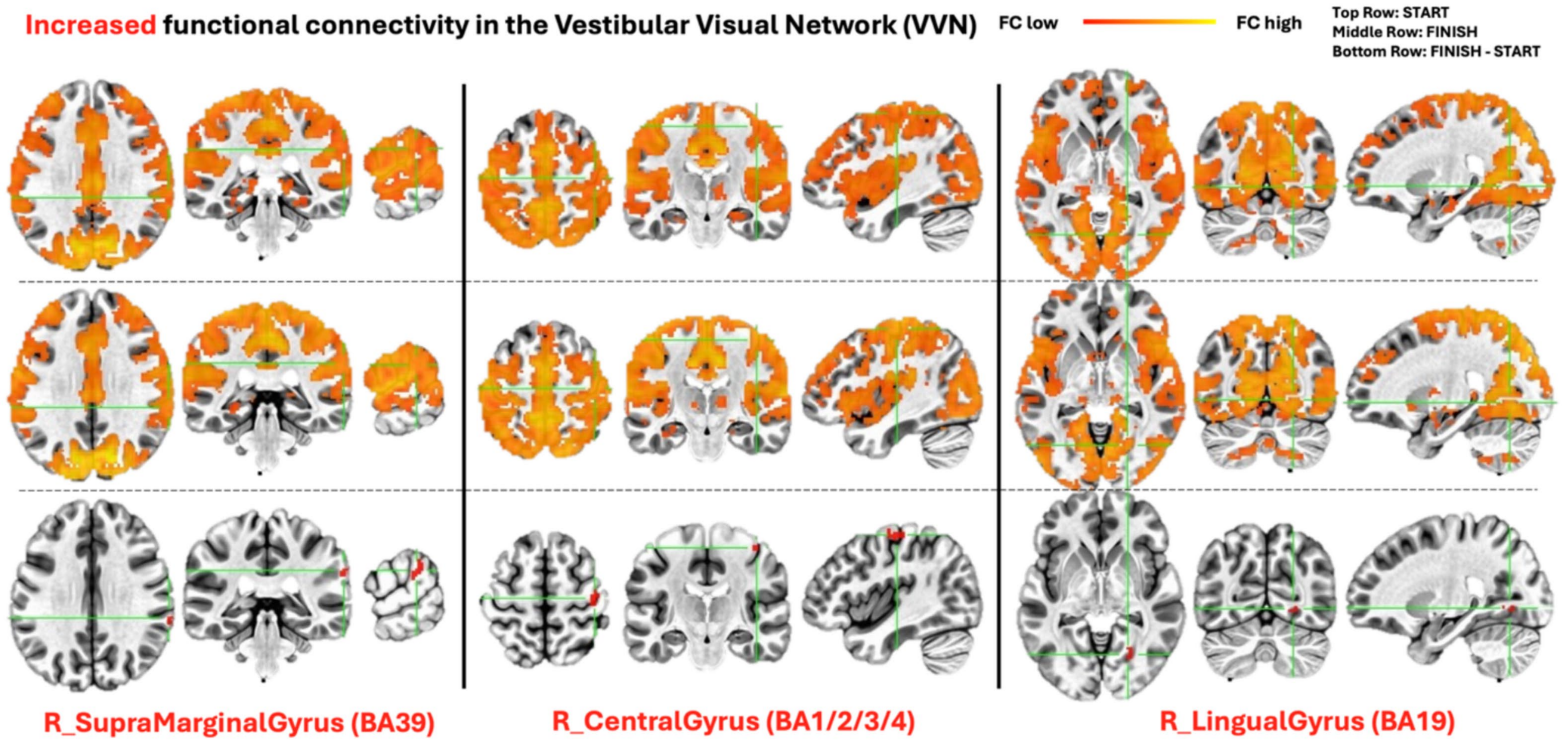
Three brain regions (the left, middle, and right panels separated by the black vertical lines) with increased functional connectivity (FC) in the vestibular visual network (VVN). Each region is shown in axial, coronal, and sagittal view in the bottom row with its location indicated by both the red blob and the green crosshair. For each view of each region, the corresponding maps of FC (*p* < 0.001/voxel plus a cluster of 172 mm^3^, *p* < 0.05 corrected) are shown in the top row for “start” (baseline) and middle row for “finish” (after 4 weeks of RehabXR training). The increased FC is demonstrated by a voxel’s color shift toward the yellow side of the color-coding spectrum. R, right; BA, Brodmann Area.

**Figure 4:**
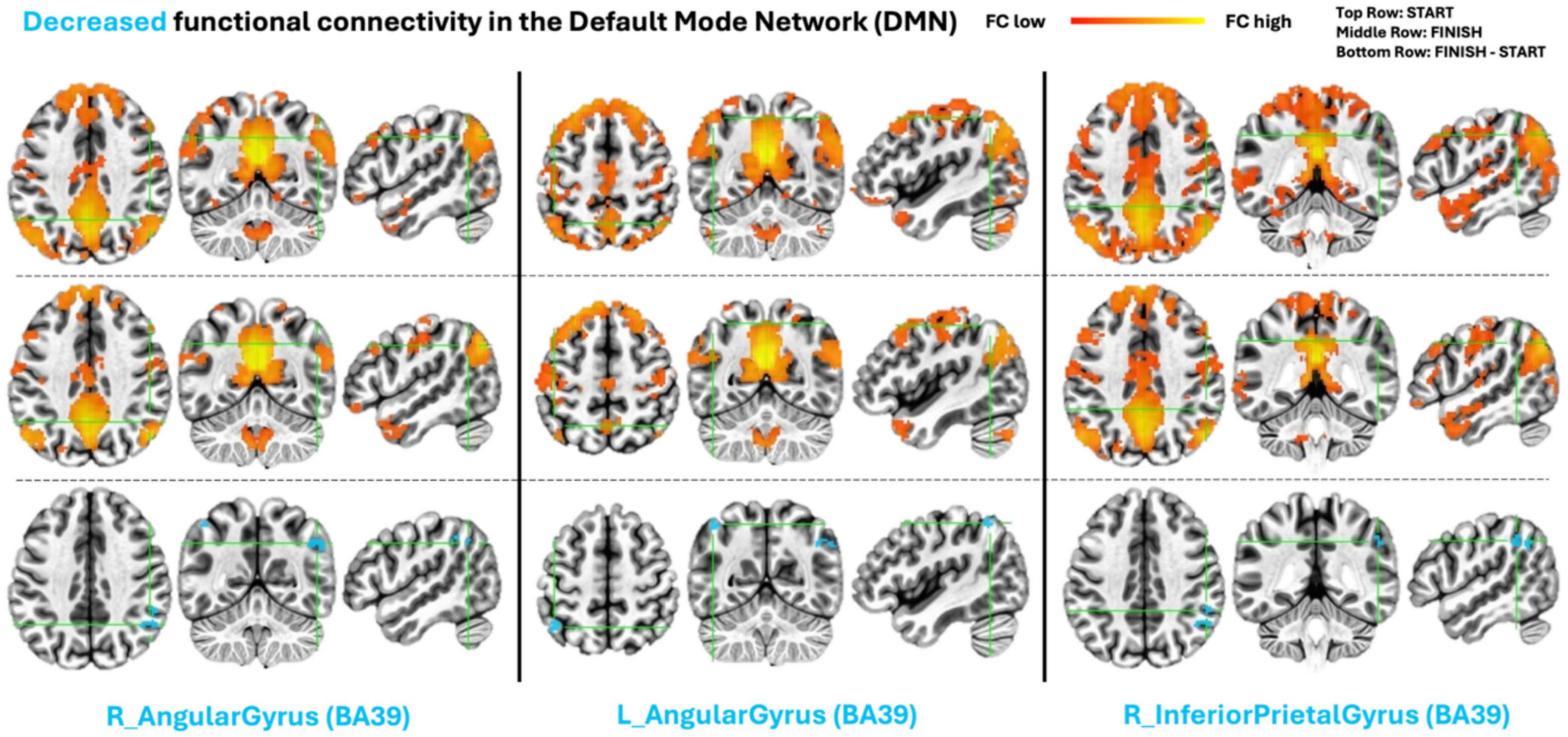
Three brain regions (the left, middle, and right panels separated by the black vertical lines) with decreased functional connectivity (FC) in the default mode network (DMN). The layout and threshold of this figure is the same as Figure 3, except that region blobs in the bottom row and associated text labels are depicted in blue, indicating decreased FC. L, left; R, right; BA, Brodmann area.

**Figure 5:**
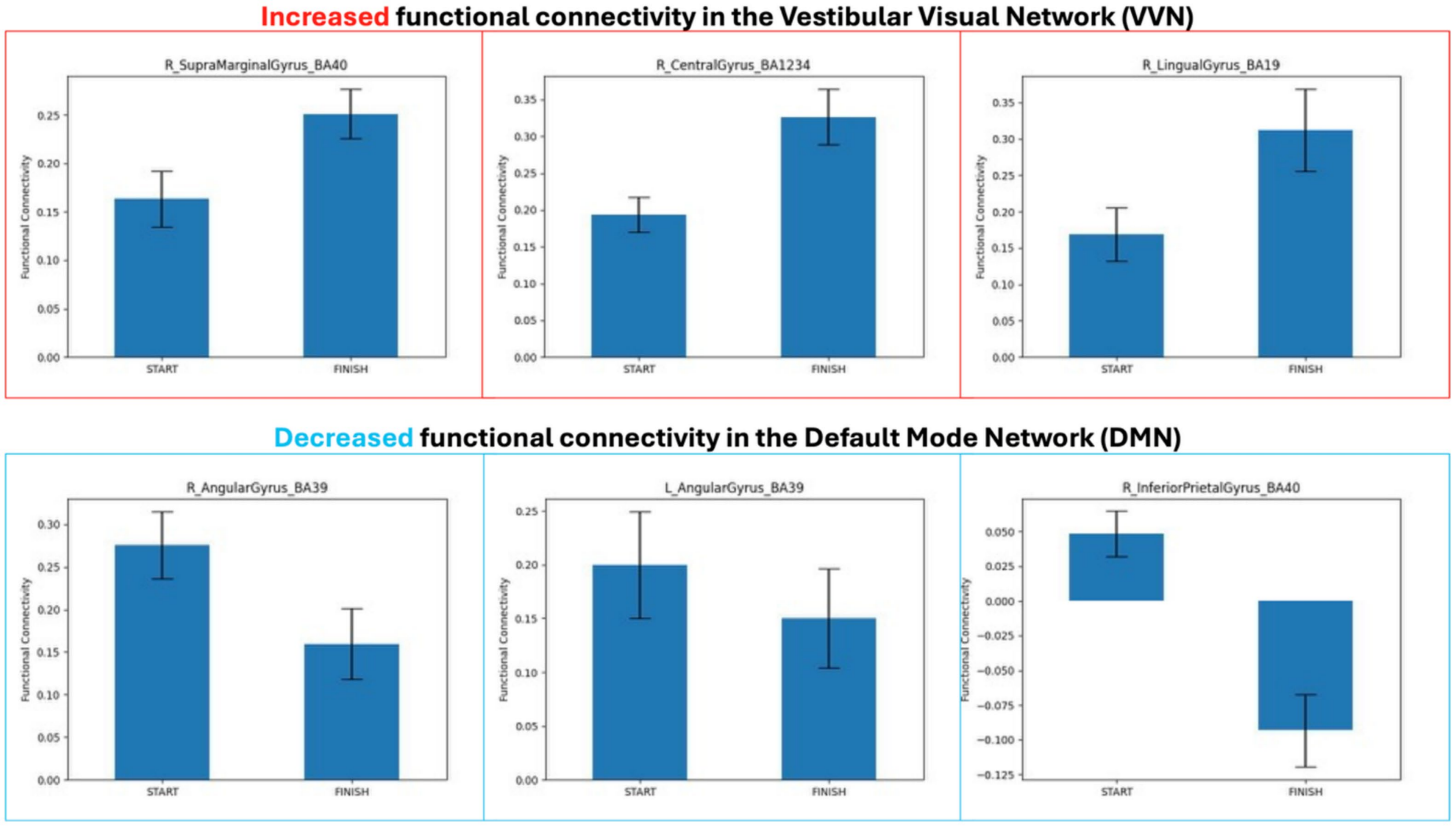
Quantitative comparison between the “start” (baseline) and “finish” (after 4 weeks of RehabXR training) in the 3 vestibular visual network regions with increased functional connectivity (FC) (top row) and the 3 default mode network regions with decreased FC (bottom row). L, Left; R, Right; BA, Brodmann Area.

The LMEM detected three regions in the VVN with increased FC, and three regions in the DMN with decreased FC (Table 3). The three VVN regions (Figure 3) are the right supramarginal gyrus (Brodmann area 40, or BA40), the right central gyrus (BA1/2/3/4) and the right lingual gyrus (BA19). These are regions underlying visual attention, sensorimotor functioning, and visual perception, respectively. The three detected DMN regions (Figure 4) with decreased FC are the bilateral angular gyrus (BA39) and the right inferior parietal gyrus (BA40). These are core regions in the DMN for self-referential processing such as autobiographical memory or internal thoughts. Besides the connectivity maps, quantitative comparisons on FC for all the detected regions are also visualized in Figure 5.

**Table 3:** Clusters showing a significant training effect in the Vestibular Visual Network (VVN) and Default Mode Network (DMN).

Functional network	# Voxels (2.5 mm^3^)	X (mm)	Y (mm)	Z (mm)	Anatomical label
VVN					
	23	65.1	−35.2	29.7	Right Supramarginal Gyrus (BA40)
	21	41.8	−19.0	59.0	Right Central Gyrus (BA1/2/3/4)
	12	23.5	−66.5	−3.1	Right Lingual Gyrus (BA19)
DMN					
	28	49.9	−55.0	37.7	Right Angular Gyrus (BA39)
	20	−44.6	−57.7	54.3	Left Angular Gyrus (BA39)
	17	52.4	−43.7	38.9	Right Inferior Parietal Gyrus (BA40)

BA, Brodmann Area. The Montreal Neurological Institute (MNI) coordinates of XYZ are in the orientation of left/posterior/inferior (LPI).

### 3.3 Correlations between FC changes in the VVN and DMN

**Figure 6:**
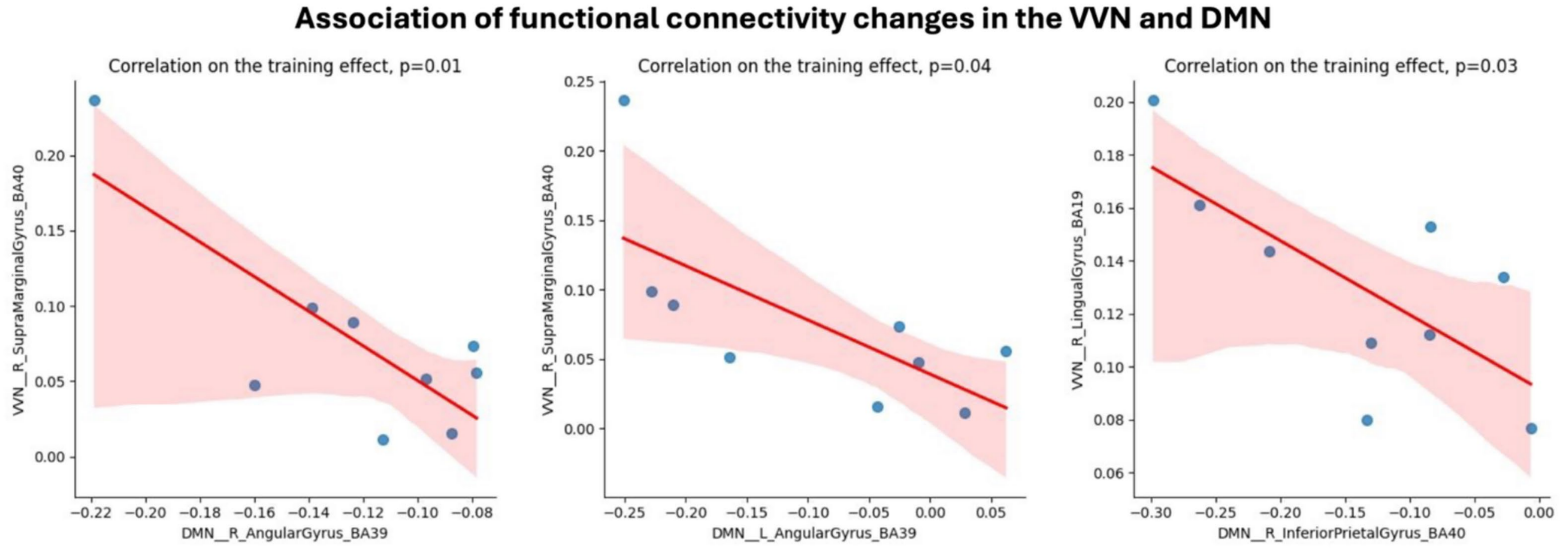
Associations of functional connectivity (FC) changes between vestibular visal network (VVN)—default mode network (DMN) region pairs of R_SupraMarginalGyrus—R_AngularGyrus (left), R_SupraMarginalGyurs—L_AngularGyrus (middle), and R_LingualGyrus—R_inferiorPrietalGyrus (right). The more positive FC changes (increases) in the VVN (Y axis), the more negative changes (decreases) in the DMN (X axis). These plots were from a subset of 9 subjects with usable data in both the “start” (baseline) and “finish” (after 4 weeks of RehabXR training) sessions. L, left; R, right; BA, Brodmann area.

Because of the competition between VVN and DMN for attention resources, when the VVN has been strengthened for improved body orientation, balance, or visual motion, the increased VVN connectivity should be correlated with decreased DMN connectivity. We tested this hypothesis, and it was indeed the case. As shown in Figure 6, significant correlations are noted between three region pairs of VVN—DMN: (i) R_SupraMarginalGyrus—R_AngularGyrus, (ii) R_SupraMarginalGyurs—L_AngularGyrus, and (iii) R_LingualGyrus—R_inferiorPrietalGyrus, where FC increases in the VVN exhibited a significant correlation with FC decreases in the DMN.

### 3.4 Correlations between FC changes in the brain and performance changes in behavior

**Figure 7:**
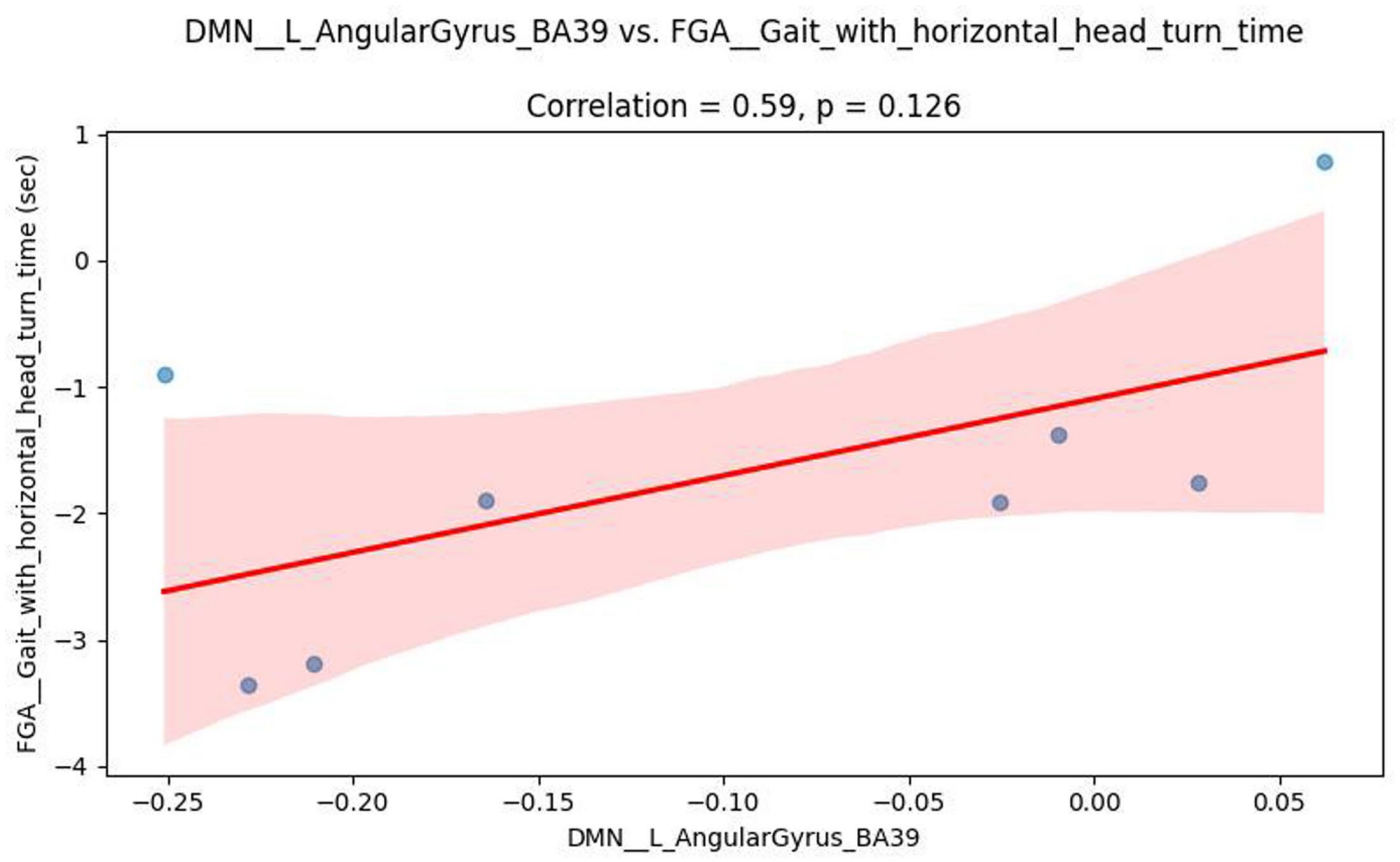
Associations between behavioral changes (“finish” - “start”) on the completion time of “FGA: Gait with horital head turns” and decreased Default Mode Network (DMN) FC in the L_AngularGyrus. Due to data attrition on both neuroimaging and behavioral tests, the overlapping sample from both sides only included 8 subjects. L: left, BA: Brodmann Area, FGA: Functional Gait Assessment.

To explore if any of the FC changes detected in the brain are potential biomarkers for performance changes exhibited in behavior, we also examined pair-wise FC—behavior correlations between the six FC changes in the brain (Table 2) and the behavioral change on “HAM-4-mTBI: FGA: Gait with horizontal head turns time”, which was specific to RehabXR. Due to the limited sample size (data attritions happened on both the imaging and behavioral test), these correlations were all insignificant. However, the correlation between decreased DMN FC in the L_AngularGyrus and decreased completion time on “HAM-4-mTBI: FGA: Gait with horizontal head turns” was noticeable (*p* = 0.13, Figure 7).

## 4 Discussion

Comparing FC measures before and after the RehabXR training, the present study examined functional brain network changes associated with this training, as well as potential correlations (with a reduced sample size) between these changes in the brain and performance changes in behavior on military-relevant tasks. The RehabXR virtual reality system was designed to deliver military-specific vestibular rehabilitation for the sensory, motor, and cognitive deficits that commonly follow mTBI. In evaluating the effectiveness of this intervention, we observed significant increases and decreases of FC in the VVN and DMN, respectively; and these FC changes are consistent with the known antagonistic coupling between these two networks. Together with behavioral changes on the Readiness Assessment Battery tests, these FC changes provide preliminary evidence in supporting effectiveness of the RehabXR intervention and suggest potential biomarkers.

The VVN, responsible for integrating balance, spatial orientation, and visual processing, is commonly disrupted in individuals with mTBI. This disruption can lead to symptoms such as dizziness or visual motion sensitivity. Following mTBI, neuroimaging studies have identified alterations in FC within this network (Slobounov et al., 2011; Smith et al., 2022; Wu et al., 2016). For instance, Smith et al. reported a diminished integration among vestibular and affective nodes (Smith et al., 2022). Additionally, in patients with vestibular migraine, decreased FC in the sensorimotor network was also noted (Li et al., 2022). The present observations of increased FC in VVN after the RehabXR training may suggest a potential remedy through strengthened processing for visual spatial information and visual motor control. More specifically, the increased FC in the R_SupraMarginalGyrus could reflect enhanced visual attention and spatial awareness that involve encoding in egocentric coordinates (Medina et al., 2009) and proprioception (Ben-Shabat et al., 2015). Meanwhile, in supporting the enhanced visuomotor functioning, the presently observed FC increases in the R_CentralGyrus (for proprioception) and R_LingualGyrus (for visual perception) also highlight the integrated processing of sensory information necessary for coordinated motor actions during simulated military-relevant tasks. Enhanced visual attention, strengthened processing for visual spatial orientation, and improved spatial awareness via rapid egocentric spatial mapping might translate into improved situational awareness, more effective Close Quarters Battle (i.e., friend vs. foe target discrimination, room entries), and more efficient urban navigation for Special Operations Forces personnel. Similarly, enhanced proprioception may provide more precise weapons handling and improved dynamic postural control (i.e., transitioning between standing and kneeling shooting positions; navigating uneven terrain or moving platforms like aircraft or watercraft; operating in low-light or darkness). This might also prevent balance-related missteps that might result in musculoskeletal injury.

Since the brain dynamically allocates attentional resources between networks responsible for processing external sensory information and internal mentation with notable antagonism (Fox et al., 2005; Raichle, 2015), the reliability of the presently observed training effect in VVN can be further justified by the other observation of decreased FC in the DMN, as well as by the negative correlations between the FC changes in these two networks (Figure 7). To overcome vestibular dysfunction, such as dizziness, the VVN needs to increase its engagement to compensate for ambiguous or conflicting sensory inputs, demanding greater attentional resources for spatial orientation and postural control. This heightened demand should simultaneously suppress the DMN, which is normally involved in mind-wandering, self-referential thoughts, and creativity. This antagonistic compensatory shift between VVN and DMN may parallel network-level adaptations described in other acquired focal brain injuries (e.g., subcortical stroke), where enhanced engagement of task-relevant networks and suppression of competing networks support behavioral recovery (Fan et al., 2026), as well as broader development changes in hemispheric asymmetry that shape cognitive outcomes (Wu et al., 2025). In other words, the RehabXR training may involve a compensatory network reorganization that biases individual’s resting attention more toward the external environment. This may be advantageous for Special Operations Forces personnel, providing them with increased attention to maintaining situational awareness in hostile environments.

Besides the correlation of FC changes in the VVN and DMN, we also noted a marginally significant correlation between the decreased FC in the DMN_L_AngularGyrus and the decreased completion time on the “Gait with horizontal head turns” task. This brain-behavior association may represent preliminary evidence of considering rfMRI metrics as biomarkers for the effectiveness of RehabXR training. Relatedly, similar results were reported in TBI patients where sustained attention impairments are associated with increased DMN activations (Bonnelle et al., 2011). With cumulated samples in such rehabilitation programs, neuroimaging biomarkers hold potential in predicting rehabilitative outcomes (Sassani et al., 2025; Swanson et al., 2025).

Despite the promising findings, several limitations should be acknowledged. First, the small sample size restricts the generalizability of our findings and necessitates caution in extrapolating results to a larger or more diverse population. This is particularly true for the present conjunctive correlation analysis between rfMRI and behavioral measures. Our limited sample was further constrained in middle-aged military males, so potential variations associated with age, sex, or civilian status remains unexplored. Second, this single-site study focused on a specialized military cohort, which may not capture the full spectrum of clinical presentations seen in broader populations with mTBI. Third, the non-randomized study design limits our ability to infer causal relationships. Without repeated neuroimaging in non-injured individuals, we cannot determine whether the observed FC changes are specific to the mTBI recovery, or instead, simply reflect non-specific influences such as scanner habituation or the passage of time. Finally, although the RehabXR group received the standardized SPaR Program plus the novel RehabXR VR-based intervention, other concurrent treatments or variations in standard care could have influenced rehabilitation outcomes. Future research should address these limitations by employing larger, more diverse samples, multicenter trials, and randomized controlled designs to strengthen the evidence base for RehabXR-augmented vestibular rehabilitation in military and civilian populations.

## Data Availability

The raw data supporting the conclusions of this article will be made available by the authors, without undue reservation.

## Footnotes

1
https://neurosynth.org
